# Treatment of Abscesses

**Published:** 1891-01

**Authors:** Frank Overholser

**Affiliations:** Logansport.


ARTICLE II.

### TREATMENT OF ABSCESSES.

BY DR. FRANK OVERHOLSER, OF LOGANSPORT.

My interest in the subject of abscess has been considerably awakened by several cases of aggravated form of chronic abscess which have come under my observation. At the same time impressing me with the fact that the dental surgeon need not complain of responsibility at times, to say the least. I am indebted to several writers for the knowledge of the different forms of abscess, especially to Prof. Gorgas. Not hoping to bring out much, if any, new thought on the subject myself, however, I shall review the subject briefly, taking advantage of the help afforded.

The term "abscess" denotes a collection of pus in the substance of the tissues, and the formation of the acute form is as follows: The exciting cause, acting as an irritant, causes an afflux of blood to the center of the affection, and the distended capillaries pour out liquid exudation, which coagulates at the center into plastic lymph, expanding the meshes of the neighboring tissues with a more serous fluid. Leucocytes are formed from cell proliferation in the plastic lymph, but on account of their not being in proper place for their growth and development into tissue they become changed into pus, which collects in a cavity formed by it, and the result is an abscess. As these steps occur in quick succession, pain, heat, redness and swelling are present with a constitutional disturbance in the form of hectic fever. An abscess without the symptoms of pain, heat and redness is termed a cold abscess, and is dependent on a low degree of vitality. The alveolar abscess is, of course, the one we are most interested in, and is first indicated by pain, constant in character, which is increased at each pulsation, then swelling about the affected tooth, which becomes more defined, and afterwards points and discharges pus, when the active symptoms subside. If, after the symptoms of pain, heat and redness subside, small quantities of pus discharge through a fistulous opening, we have the chronic form of alveolar abscess. The location of alveolar abscess primarily is the apical space wherever it may afterwards extend. The manner in which the pus leaves its bony enclosure largely determines the difficulty of treatment. It may at once penetrate the soft tissues, which is the simplest form.

After the pus reaches the soft tissues the pain is greatly reduced, and the pain changes rapidly to swelling, sometimes distorting the face to a marked degree. This can be greatly reduced, and often wholly prevented, by the use of the lance, thus avoiding the second form, which forces the tissues of the gum from the bone and forms a cavity for itself between the two, or allows the pus to gain vent at the margin of the gums. This is the condition most favorable to necrosis, as the periosteum of the bone is raised to allow the pus to flow from the bone. In this condition the bone is deprived of its natural supply, and the result is death to that part. If the pus can be promptly discharged, however, not much harm will follow. This form of abscess in the lower jaw is more likely to point on the face than any of the acute forms. The same condition in the upper jaw, though less frequent, may point almost anywhere on the face after the pus has gained some headway in the soft tissues; since it follows in the direction of the least resistance, it may follow the course of a muscle to a surprising distance, thus pointing far from its source.

The third form is that in which the pus, instead of penetrating the surface of the bone, finds its way along the side of the root, following the peridental membrane to the margin of the gum, and is discharged at that point. When this occurs it destroys a large portion of the peridental membrane, after which the overlaying alveolar process quickly disappears. This can be recognized by passing a thin, flat instrument along the root of the tooth. Teeth suffering from this condition are most likely to lose their attachment and almost drop out, depending of course on the extent of the destruction of the peridental membrane.

In the treatment of any form of abscess, the first and most important point is drainage, to carry off the pus. This should be anticipated early if the abscess has not already pointed, that the location of the opening may be made at a proper point, thus avoiding an opening on the face. The irritating cause should be removed, which, in most cases, is a putrescent pulp, or defective root filling, which may prolong the disease indefinitely, as chronic abscesses very rarely cure themselves in any part of the body.

The chronic form of abscess usually follows the acute if left to take its course, as the conditions that produced it are present to continue its supply. A putrescent pulp left in a tooth may continue its discharge of septic matter into the apical space indefinitely, thus necessitating a constant discharge through the fistulous opening, or if the fistulous opening closes up, the pus may form a cavity in the tissues and remain unobserved except a little soreness occasionally until extensive caries of the bone has been suffered. Certain forms of cold or blind abscesses are sometimes overlooked or difficult to diagnose. "Fluctuation," by pressing the fingers gently over the affected part in such a manner as to allow the pus to pass under the fingers, gives umistakable evidence of pus formation in such cases.

The discharge of the pus is the primary object in treatment. This is usually easy of accomplishment, except in certain blind abscesses or where extensive caries of the bone has taken place, forming a cavity in the bone. Once opened, it should be kept drained if the tendency is to close up. This can be accomplished by a cotton or wool tent twisted to proper size and saturated with some disinfectant, preferably of an oily nature. The pus will usually ooze out through the tent. If not, it can easily be removed, and thus avoid a second lancing. Some of the accepted disinfectants, such as bi-chloride of mercury, peroxide of hydrogen, oil of eucalyptus, phenol or camphol, phenique, should be freely used with a hypodermic or other medicinal syringe. If any necrosed or carious bone is found it should be freely removed. It is remarkable how quickly new bone structure will form in the maxillary regions if nature is only given a chance. Abscesses are often treated too long before surgical means are resorted to. We might as well extract the tooth at once as to attempt treatment with necrosed or carious bone surrounding it. If the cause is present to feed it we can not hope to cure it. There is often much larger destruction of bony tissue in a chronic abscess than we are aware of, and simply forcing a pledget of cotton containing a disinfectant into an abscessed tooth is insufficient. The medicaments should pass through the whole course of the abscess, and this can only be accomplished by the use of the syringe. Peroxide of hydrogen is the best remedy in pus formations, as it has a perfect affinity for pus and destroys it. In the use of peroxide of hydrogen, it should be used until effervescence ceases. After all septic influences are removed and the parts kept free from such influences, nature does rapid work in healing.

With your permission I will speak of an aggravated case of abscess that came under my treatment. A young man of twenty-two years of age came to me for the treatment of an abscess that originated from a second molar. The tooth had been extracted four months when the case came to me for treatment, but the abscess continued. The pus had discharged all this time through a fistulous opening on the check, having destroyed large portions of the underlying tissue. The salivary duct had either been cut in lancing or was destroyed by disease, no doubt the latter. This discharged the saliva on the outside of the cheek, and flowed almost all the time. This was a most discouraging feature of the case. After making an incision on the inside to meet the remaining part of the duct, it would refuse to flow through its new opening into the mouth, but constantly gained vent on the cheek, and was only thrown into its natural channel by a silver tube being introduced at the natural opening and plastering up the outside escape with adhesive strips. The pus had three openings on the face, and was not able to direct its course to the inside, owing to the reduced vitality of the outer surface; it would always gain a new opening in the weakened tissues. The difficulty was caused by extensive caries of the bone, on which I

operated three times before removing sufficient bone to give a new start to the healthy tissues. The peroxide of hydrogen was my principal remedy, and I sometimes used two ounces before the effervescence ceased. Other remedies were used, but not so satisfactorily. The case healed nicely, and aside from a scar on the cheek the young man suffers no inconvenience.

DISCUSSION.

Dr. Hunt. The doctor has given us a very interesting paper, and certainly the case that he describes is very interesting. I will ask the doctor, what became of the severed duct ? Was it established again ? Did it perform its functions ?

Dr. Overholser. Yes; that was my object in introducing the silver tube. The tissues had so wasted away that it would not pass through the old duct, and I perforated that and connected with a silver tube, and after a time it seemed to fill in around that.

Dr. Hunt. Forming a new outlet ?

Dr. Overholser. Yes, I had some difficulty in getting it through. The main difficulty was in keeping the saliva from running down on the outside of the face.

Dr. Hunt. The ordinary abscess that the dentist meets with I think is that requiring treatment by drainage and the removal of the original cause of the abscess; and in a large majority of cases we know that that is all the treatment necessary. Even in those cases where it comes from the tooth (that is an abscess as we consider it,) with the removal of the fetid matter in the canal of the tooth and the healing of the part and filling it, without any further treatment, we will have a cure and establish good health.

There are other forms of abscess. The blind abscess —I think it is most easily cured, and the quickest way is to tap it from the alveolar process near the apex of the root, after which you can bridge, or drilling through the end of the root. I have had very good success in doing that. I know that there are some operators that greatly prefer going through the outside, and I think that is necessary to a cure in those cases where you must evacuate all the pus before you can hope for a cure. I think the doctor covered the ground very thoroughly, and I do not now think of anything to add.

Dr. Wilson. I think the essayist is right in saying that we very often learn as much from a discussion as from a paper. I enjoyed the paper, and I think he has been remarkably successful in that operation that he speaks about, because I do not think that is a very easy thing to accomplish. There are certain classes of those cases that bother me very much. In a case of what you might call a chronic abscess, where the tooth probably had been filled for a long while, and there had been a little fistulous opening that did not yield to treatment, I simply began to remove the cause of the dead pulp where there is an opening into the pulp cavity, giving the abscess opportunity to secrete pus. While some men may be able to treat that abscess by immediate root filling, in my office it does not work very well. I think it needs something else besides simple treatment, and as Dr. Hunt has intimated, I think in some of those cases that there must be something else done. In many cases of that kind I think you will find the ends of those roots denuded of any periosteum. Very often there has been a little absorption, and after extracting the tooth you will find the end of it sharp, so that if the soft tissues had come against it, it would have irritated them, and I think you have got to do something else than to put medicine into the root or fill the root. I think you will never treat the tooth successfully so that there will not be any abscess there, or any pus secreted, unless you smooth off the end of the root in some way. I do not say that I do that in every case, but I have done it in a few, because I do not like to undertake to treat an abscess of one of the incisors and not be able to stop the formation of pus. I do not want to see it in two or three months from that time with a little fistulous opening there. While there may not be much pus there, I know there is a little pus formed, because of the fistulous opening. Those cases I find hard to manage. While some people can cure them, I do not think I can. I think I can if I cut down and smooth off the end of the root and protect those tissues so that there will not be new tissues formed there. But abscesses of that kind I think are hard to handle.

Dr. Hunt. I had a case sent to me within the last three months of a tooth that I replanted nineteen years ago. I was glad to see the old fellow again. A number of years after it was replanted I took out and dressed off the ends of the roots, as all of you, I guess, have done. It remained there for a number of years, but I can not tell how long, before the abscess recurred. It did come back, however, and he wore the tooth for several years before it finally came out. He had it removed, not from the pain, but because the tissues were absorbed all around and it became an annoyance. It was a lateral incisor. I guess Dr. Hacker remembers the case.

Dr. Hacker. Yes, I remember it.

Dr. Wells. Had there been much additional absorption ?

Dr. Hunt. There had been considerable on the end where I had smoothed it off years before and polished it. In the early days of replanting I think I met with a good many failures for want of antiseptic treatment. We did not understand it as well then as we do now, and a good many of these operations appear to me more successful to-day than fifteen or twenty years ago, on account of our better knowledge of antiseptics and their use. I remember replanting a tooth that a man had carried in his vest pocket, I think, two days. I simply washed it off and replanted it for him, but when I put it in there it suppurated most violently, and dropped out. It would not unite at all, and I think that if I had that tooth to-day I could make it unite and be of service to him.

Dr. Wells. While I was very much interested in the paper, and I think it covered the ground pretty thoroughly, I would like to know how to diagnose a case thoroughly. I believe it is considered sufficient evidence if we find a fistulous opening opposite the root. Isn't that so considered?

Dr. Overholser. Yes, that is an alveolar abscess, and usually in a chronic form.

Dr. Wells. I had a case come under my observation of an abscess that was chronic, and had been discharging for a long time right over the root, and I usually conclude in such cases that there was an abscess resulting from the death of the pulp. In order to remove the cause, I proceeded to drill the tooth and take the filling out, and I found that tooth alive. What kind of an abscess was that?

Dr. Overholser. That was an abscess, possibly, of the very character that I spoke of, on which, for some reason or other (possibly from calcareous deposit destroying the peridental membrane) it had created a pus formation that had to be discharged at the margin of the gums, thus destroying the vitality of the peridental membrane, and not destroying the pulp. I would say that that is not properly an alveolar abscess; that is an abscess, but the proper place, the primary position for an alveolar abscess, is the apical space at the very apex of the tooth; and properly speaking, an abscess has its origin any place, else it is not properly an alveolar abscess in the best sense. But no doubt something irritated the membrane. It might have been regulations in early life, regulating the tooth, or some blow on the tooth that destroyed the membrane without strangulating the pulp, possibly.

In regard to what Dr. Wilson said as to the difficulty of treating some teeth, I have no doubt but all wise men will come to the conclusion that we can not save all teeth.

There are teeth of that character that come under that very head, where the peridental membrane is destroyed, with possibly considerable absorption of the alveolus, that is not in shape to hold the tooth, and if we conclude that it would be better to do as Doctor Hunt says, extract the tooth, remove the diseased bone, and put it back again, but that, of course, you know, is an uncertain operation, and does not always succeed. We can not hope to save all teeth. Sometimes it is a very difficult matter to cure an abscess, let alone save the tooth. Usually we can cure the abscess, but there is always more or less carious bone surrounding an abscess, and the vitality of the immediate parts is lowered to a great extent. It will occasionally slough off a little, and become very offensive for a long time, and there are some cases that we can not hope to save.

Dr. Hacker. I remember very distinctly the case that Dr. Hunt speaks of, about nine years ago. It was the first case of replanting I had ever seen. He gave me part of the work. I dressed the roots of it before replanting. I remember it very distinctly, and following that up, I became very enthusiastic over the removal of teeth and replanting. I followed it up very successfully, and do it yet. I think it is the surest means of stopping an abscess on the molars and bicuspids, and I have tried every way that I ever heard or read of. I would almost undertake to say that if you can make a good root drainage, you can save probably nine out of ten teeth by replanting. I had one case of a right lower molar about six years ago. The patient went to Chicago, and Dr. Harlan wrote me for a history of the case. I told him what I had done. He removed the tooth and put it back again. That was about four years ago. The abscess got well, and that was a tooth that had been replanted a second time. That you can call heroic treatment, but it is as much a success as treating an abscess through the tooth, or through a fistulous opening.

Dr. Oliver. I think Dr. Overholser deserves a great deal of credit for his paper, from the simple fact that in the case he cites nine out of ten dentists would have failed to save the tooth. Dentists, as a rule, have great hesitancy in undertaking such cases, when they really know more about it and are better able to operate than any physician, and they know more about the organs of the mouth and the head in general than the average physician, and there is no reason at all why they should not attempt such operations. I think he cites the case of an abscess that formed after the tooth was extracted. Was the abscess there before —and then the tooth extracted—and the abscess returned?

Dr. Overholser. The abscess remained after extraction. That is frequently the case. I had something like that kind of a case come into my office about three weeks ago, where the patient said the tooth was aching, and there was nothing at all the matter with the tooth. It was in the second superior right bicuspid, and there was a large abscess which extended, I thought, into the antrum. There was quite a good deal of inflammation there. Two weeks ago last Sunday, with the assistance of Dr. Cross, I operated on him, and I succeeded in removing a piece of necrosed bone about the size of a butter-bean and very much in that shape. I got the dead bone out of there and treated it afterward for ten days with nestorine. I found nestorine one of the best antiseptic solutions that can be used. Nestorine has a great deal of drastic qualities in it. It was a complete cure after ten days. I saw the case Saturday and there was nothing there but a scar on the gum.

Dr. Oliver. I would like to ask Dr. Overholser what he would do in the case of an antrum abscess, an abscess either of the bicuspids or molars and afterward filled with pus? I would like to have light on the question as to the right way of treating it. I have had such a case recently.

Dr. Overholser. The principal difficulty in treating an abscess of the antrum is to give vent to the pus. Of course, if the pus has remained there a long time and becomes very offensive and unhealthy, it might destroy the membrane to such an extent that it would become serious enough to remove the floor of the antrum. But usually if it is affected and the abscess is really caused from the tooth, the extraction of the tooth and drilling through the lower floor of the antrum will allow the pus to escape, and the antiseptic washes that we have, such as nestorine and the phenols and mild carbolic acid wash in such cases, are about the best to use. I have had several cases of antrum difficulty. They are oftentimes seemingly the outgrowth of catarrh, in which the membranes have become disintegrated more or less through the catarrhal affections, and that is caused probably by the fact that an unhealthy condition of the membranes is produced and the opening is so small that they are swollen shut, and it becomes offensive from the lack of ventilation. As soon as ventilation is brought about the disease is likely to subside. I have usually extracted the tooth, though sometimes it has been in above the tooth in the antrum.

Dr. Brown. We have the benefit of having with us a newly-acquired member, who has distinguished himself in the treatment of the antrum. He has this last month effected a cure where an oculist of considerable reputation had failed, in a case where the orbit of the eye was involved. He is present with us this evening, and can give us a great deal of important information. I know his practice is distinguished. His name is Dr. J. D. Coyle, of Fort Wayne.

Dr. Coyle. Gentlemen, I could not address you; it is out of my line of business. I have succeeded in practicing, but I really could not speak. I am a self-made dentist, with no instructions from any one outside of my own practice, and I am not able to speak.

Dr. Overholser. Will you tell how to treat an abscess ?

Dr. Coyle. If you want to know my treatment, aromatic sulphuric acid is my main remedy.

Dr. Overholser. How do you apply it?

Dr. Coyle. I apply it with a hypodermic syringe.

Dr. Hacker. Through what kind of an opening?

Dr. Coyle. It is always an opening with an instrument. If there is not an opening, I make one.

Dr. Hacker. Where?

Dr. Coyle. In the most convenient place. By removing the tooth usually.

Dr. Hacker. Do you usually make more than one opening?

Dr. Coyle. Usually two.

Dr. Hacker. I have never found it necessary to make more than one opening.

Dr. Coyle. In twelve years I have had fifteen cases. One case was perfectly plain. It had been treated by Terre Haute and Indianapolis oculists for six years, and they never discovered the difficulty.

Dr. Hacker. Do you generally open up through a bicuspid or a third molar?

Dr. Coyle. I have had more cases that are caused by the second bicuspid than by the first or second molar, or even the third. I had a case this spring. It was a tooth that was filled twenty years ago, and it seemed that for twelve years it had been paining him a little. I made an examination and removed the tooth. I told him before I removed it that I thought the root was destroyed and found it so and removed it, and the instrument broke off in the root. That still remained, but the root was absorbed away from it probably a quarter of an inch. I had easy access to the antrum, and treated it six weeks, and pronounced it well.

Dr. Hacker. In what condition did you find the antrum after taking out the tooth?

Dr. Coyle. I could not explore it from the third molar, any further than just the socket there. Of course I found the bone slightly carious. That I could discover with the probe.

This recent case that I had was that of a merchant of our city. About three months ago he was attacked with a hot water discharge from his eye. It would run down so hot that it scalded his face. He went to our best oculists. They treated the eye directly. I do not know what remedy they used. They treated the eye, and also wanted to operate on the tear duct for the discharge of pus. When he came to me he was wiping away a tear every minute or two. I made an examination, and found that in the second bicuspid that I had filled some six or seven years ago the pulp had died in the meantime. I do not know why, but on percussion I found that that was the original cause of trouble, and told him so, and removed the tooth, and found the root absorbed, and a little fungus growth came with it. I could not get access to the antrum, but introduced one drop of aromatic sulphuric acid, pure, with my syringe, and cauterized that sac. I have had three cases without boring the floor of the antrum. I use diluted sulphuric acid, ten to one. That is only one case in several that have come through the hands of physicians who have treated them for months.

Dr. Overholser. What symptoms do you regard as the most reliable in diagnosing a case?

Dr. Coyle. Usually in the morning when they rise and blow their nose, there will be a little drop of blood come from the side of the nose. That is one. In women and all delicate people there will be a little pink flush probably over the antrum. But you will never find two cases alike; I am sorry the doctor called your attention to that, but when you were speaking of it I was anxious to hear you gentlemen and old practitioners and educated men talk about the matter. I have never had any opportunity to hear anybody on the subject.

Dr. Overholser. These cases had never been treated until you treated them?

Dr. Coyle. They had never been treated—you mean after they were treated by these eminent men?

Dr. Overholser. Not by dentists?

Dr. Coyle. No, they were mostly general practitioners.

Dr. Hunt. As to the subject of diseases of the antrum, I think we very often have slight diseases of the mucous membrane of the antrum when we do not know it and the patient does not know it, and we know that the roots of the molars and the roots of the bicuspids frequently make an impress in the floor of the antrum. Now, when those roots become affected, and affected with abscess, is it not very surprising that we do not have more diseases of the antrum than we do? As I said before, I think there are a great many slight inflammations of the mucous membrane of the antrum that we nor the patient are not aware of. The treatment, of course, will be to establish a drain the first thing. The outlet into the nose being above the floor of the antrum, it does not give a very good drain, and if it ceases the disease is established there. But by the removal of the tooth you can generally get into the antrum through the alveolus. If it is necessary to open it, of course open it until you can get in through the bony tissue, and if there is necrosed bone there you can generally distinguish it with a probe. It is easily detected when you come to it, and if there is no necrosed bone the cure will very frequently be effected by simply establishing a drainage through it a little while without any further treatment. But if there is necrosed or carious bone there you must take it away before you can hope to get a cure.

Now, as to the introduction of aromatic sulphuric acid, if there is not a great territory involved in the disease, remove the carious bone and you will make a cure without an operation further than the opening into the antrum. I just dismissed a few days ago a case that was sent to me from Chicago, being that of a lady who removed from Chicago to Indianapolis. Her case appeared to be very aggravated when she came from Chicago, but we found after the first examination that it was complicated with a very considerable disease of the air passage of the face, extending from the antrum. The abscess on the end of the root was evidently the cause of the disease of the antrum, and the tooth had been removed in Chicago, and had been treated some weeks, and still there was not much improvement, so the patient said. Dr. Swain, who had charge of the case there, gave me a history of what his treatment had been, and the lady, I think, was under treatment at Indianapolis only a short time, probably ten days or two weeks, when we got the discharge from the antrum stopped entirely, but the trouble seemed to be in the frontal sinus and the passages therefrom. She had a weeping of the eye on the side on which the antrum was diseased, the fluid running down out of the eye onto the cheek and scalding and blistering the cheek, and all those symptoms which are so distressing, but under the treatment of aromatic sulphuric acid it yielded very kindly.

I have in my hand here, handed to me by Dr. Chappell, a little vial containing some specimens of teeth. He did not say what they were, but I take them to be replanted teeth. They have the appearance of having been absorbed, some of them, as deciduous teeth. Now, whether he proposes to exhibit those to prove that the operation of replanting is a failure, I do not know, but I have seen just such cases as these from replanted teeth and from transplanted teeth. And yet I do think that in many cases the operation is one that ought to be resorted to rather than suffer the loss of the teeth. We ought to take the chances, and if it succeeds is it not better than to remove the teeth entirely and put in a plate or bridge or otherwise? I think it is if there is a hope or chance of saving it by that method. I would rather take the chance, even though it should be a failure, than remove the tooth entirely, for fear of such results as that.

Dr. Chappell. On the subject of the doctor's essay, I always feel as if it was a duty that we owe to each other not to criticise. But in all our expressions, physiological or pathological, we should keep close to the text, so as to convey the proper meaning. The first thing my attention was called to in his essay was the fact that pain turned to swelling, if I did not misunderstand him. Pain, heat, swelling are symptoms of inflammation, but not the physiological phenomena leading to inflammation. Now, it is true that in the pain of the tooth, the parts being so confined, it would be more painful coming into the soft portion where there is an expansion of tissues, and if there was any determination of blood there and separation of the elements there would of course be that phenomena, and would be less pain when the swelling commenced.

On the question of the phenomena of an abscess, I differ somewhat with him in regard to describing the phenomena leading to an abscess. As to the putrescent pulp being the recurrent cause of the abscess there, I think that is a question.

There was another expression, in speaking of an abscess having a fistulous opening. An abscess ceases to be an abscess whenever any drainage commences. Then it becomes ulceration. Whenever there is a fistulous opening from an ulcerated peridental membrane, it is no longer an abscess; it is an ulcerated peridental membrane, with an alveolar fistulous opening. A blind or cold abscess is what we generally know as an abscess that has its opening through the canal. It has some microscopical drainage A cold abscess is not, as I am inclined to think, a modern way of expression. The caries and necrosis of the apex of the root, sanguinary deposit on the denuded portion of the tooth that Brother Wilson speaks of, is one cause of chronic ulceration of the peridental membrane. There are a great many gentlemen, in the clinical part of the discussion, who have great success in the treatment of abscesses, after they have formed an artificial opening, by the use of gutta-percha; even in the fistulous opening of an ulcerated peridental membrane, wherever there is a possibility that there is an absorption of the apex of the tooth, and some of the sanguinary deposit around near the line of life of the tooth, by floating or flowing of the cavity with liquid gutta-percha, even until it comes out through the fistulous opening. What becomes of it? There will be a certain amount of the flowing gutta percha that will be deposited around the end of the root, and a cystic membrane will be formed. A cystic membrane is just as much tolerated within the body of the tissues as almost any other membrane is tolerated. I think that in the case the doctor speaks of, where it comes through the cheek, there was more or less ulceration there of the salivary gland, was there not?

Dr. Overholser. I do not think it would interfere with the gland, because the gland would be back under here (indicating with finger), and this difficulty was away down in the cheek, and came from the second molar.

Dr. Chappell. A superior molar?

Dr. Overholser. Yes, sir.

Dr. Chappell. In making its track, it cut down into the muscles, didn't it?

Dr. Overholser. It got down to the muscles of the cheek.

Dr. Chappell, Wouldn't it have remedied that by making an incision and making a new track right along by the alveolar border?

Dr. Overholser. That is what I did.

Dr. Chappell. There is where you made your drainage?

Dr. Overholser. Yes.

Dr. Chappell. And then you had the wound on the cheek. Did you get any cicatrix there?

Dr. Overholser. No, it is remaining in that condition. The vitality of it was so low that it certainly would not have been wise to destroy it. It only hung over the cheek, and I was afraid to leave it there.

Dr. Chappell. Dr. Bell and Dr. Hunt spoke of cases of catarrhal trouble. There are a great many cases of catarrhal trouble of the antrum that no doubt is there at the time it gives its trouble, and we have a great many cases of perforation of the floor of the antrum and caries of the walls or aperture. Now, we know that whenever the calcific granules or ossific granules, either one—wherever there is caries, and of course wherever we have necrosed bone we have more or less carious bone, and of course the operation of cutting out there is nearly always a disfigurement. I have a case now of a patient that gives trouble. The second bicuspid is gone and the entire cavity is open with more or less ulceration. I find that drainage does not stop it, and that the vitality of the patient is such that I have not much hope of recovery. Your cases, doctor, (addressing Dr. Coyle), I believe, were where the patient was in reasonably good health, were they not?

Dr. Coyle. Some were, and some not. In one case I spoke of, a Sister of Charity, her flesh was very much reduced, and she was in a very nervous condition, so that she had to be helped up the stairs. After she was restored she regained her flesh, and she is now as healthy as ever in her life.

Dr. Chappell. We do not pay enough attention in the treatment of chronic ulceration to ascertain the fact whether it is only the drainage from the antrum.

As to these teeth that I have filled here (exhibiting specimens), out of forty-three replantations these are the only failures that I have had) these are the only ones that came back to me. I do not know how many the rest of the dentists had. These cases, we found, with the exception of the bicuspids, were the result of injury. That bicuspid stayed in until the gentleman spent a term in Princeton College, and after he graduated and came back, says he, "Doctor, I have the finest little specimen here that you ever saw in your life." I said, "I have been afraid of that specimen," and he simply took that tooth out and showed it to me and he wanted me to look in his mouth, and there was the nicest little cavity where the tooth came from. He put it back again, and went on wearing that until at last he got to taking it out and putting it in so often that it would not stay, and he came to me, and the membrane lining of the cavity was as nice as any mucous membrane. These others were the result of injury, and then secondary restoration took place.

There is restoration. There is no doubt about that. In this case here the party came to my office with a very sore front tooth. The gum was blue, and he said, "It has been giving me a good deal of trouble. It is very loose, and I would like to have you do something for it." I examined the tooth and told him it was quite loose. Says I, "I think the best thing to do for that is to take it out. Let me take it out." I just took hold of it with my fingers and pulled out the tooth, and that piece of bone with it. It fitted the tooth which you see here. There is a pivot tooth there which had been worn in the mouth two years, which had been done at Washington, Pa. The tooth had become inflamed, and the processes had become carious and necrosed, and an entire piece of the outer wall exfoliated, and all you had to do was just to take it out. I cured it in a few days and put in a plate. That is simply a reminder to be very careful when you are setting a pivot tooth not to go through the wall.

Dr. Overholser. In answer to Dr. Chappell's objections to my paper, I labor under one disadvantage, and that is that I trusted a little too much to my memory in preparing the paper, as I have written more than half of it since I arrived here to-day. But I believe that Dr. Chappell will not find the text books to substantiate his statement that an abscess is no longer an abscess after it has discharged pus in a fistulous opening, but becomes ulceration. I am confident of the fact that that is the true form of chronic abscess. Where it first forms an opening on the outside of the gum and relieves itself, it is an acute abscess; where the flow continues, it is a chronic abscess. And as to my statement of the pain changing to swelling, I did not wish to express that there was any action there that changed pain of a material character into swelling of a material character, or that one was transformed into the other. It may be that I did not clearly express myself in the paper, but I meant that where we have pain as the pus is working through the alveolar process, when it strikes the soft parts the symptoms, not the pain, changed into swelling. Dr. Chappell says that at this point we find increased pain. That, I think, is an error.

Dr. Chappell. No; swelling when the pain ceases.

Dr. Overholser. That is the statement I make in my paper, and I understood you to say the reverse. The idea is that when the pus passes through the bony tissue and gets into the soft tissue, there is so much resistance and secondary swelling, and as soon as it stops swelling it ceases to pain. That was my experience.

As to the other techanical terms in the paper, I might have expressed myself a little more in accordance with the text books. The terms selected were just as they came to my mind, because I was away from my text books. But I do not believe that they were strictly unprofessional expressions. I thank Dr. Chappell, however, very kindly for his criticism, because it is a subject that I am interested in, and I like to see it brought out, and a friendly discussion and criticism makes us all better men.

Dr. Chappell. I did not wish to find fault with the gentleman, only that I wished to criticize in a general way the expressions. You will notice some expressions in our text books are the same expressions that were made thirty years ago, and if you take some of the advanced writers in the general text books over the country you will find that these are rather more critical than some of our text books. Now, there would be no necessity for revision of our text books if it was not for the correction of errors. Those of you that have been writing for years, when you go to a text book you will very frequently find the same thing copied from one book to another, and it is not satisfactory, and by reading a great many on the same subject you will find once in a while an author that comes out plainly and is very technically expressed. I know that some of our text books, on the question of abscesses, treat an ulcerated peridental membrane as an abscess, and yet they are speaking clinically and plainly instead of technically and didactically.

Dr. Overholser. How would you define a chronic abscess, or wouldn't you allow a chronic abscess to exist?

Dr. Chappell. A chronic abscess is not, critically and techanically speaking, an abscess. Why? If you will take all the authors, I think they are generally settled down on the definition of an abscess as a collection of pus circumscribed by a wall (or a solid wall, as some put it) and effusion. Now, to begin with the question of irritation and determination of blood, stasis and the separation of the elements of the blood, the formation of effusive granules and the changing of the leucocites or white blood corpuscles into pus corpuscles, we have then in some way the metamorphosis of the tissue—tissue changed into pus. There are so many things that might be said on the subject, but I do not wish to take your time.

Dr. Wells. I would like to ask Dr. Chappell how many authors he has consulted that give the definition of abscesses that he has given us?

Dr. Chappell. I think I have possibly a dozen or more, that is, on the same line of treatment.

Dr. Overholser. I think if there is anything to be lamented, it is the technicality of our profession. I am a subscriber to four or five dental journals, and I find a great diversity among good writers and good thinkers, as to the language of technicality, and sometimes I think we (not in this case, Dr. Chappell), as a profession, belittle ourselves by dabbling into a thing so fastidiously that we can not find terms to express it. For instance, when we made the change from nerve to pulp. One of my journals speaks of the matter of changing the name from nerve to pulp, and what an unfortunate thing it was, since pulp expresses nothing, as it were, only a mere body, protoplasm, or something of that kind, and as we are working on a patient's tooth, and he says: "You are striking the nerve, aren't you?" "No, no; that is not the nerve." "Well, there is feeling there. Isn't a nerve the center of sensation." "Oh, yes, but that is not the nerve." Then we go a little further, and then we get into the nerve proper, and give considerable pain, and that still is not the nerve, but is pulp. The active part of the pulp is nerve structure, and it seems to me, after all, that nerve is a much better name than pulp, because we are dealing with the nerve. I am getting tired of telling patients that "That is not the nerve, but it is the pulp; that that is not nervous sensation, but is pulpitis, or something of that kind."

Dr. Chappell. Pulp sensation. Still we know we are getting into the dentine. The dentine is very full of sensitive fibrils.

Dr. Overholser. The whole tooth is ramified with some nervous tissue, or transmitting power, and it certainly must be something that transmits pain to the nerve centers, and must be a nervous force. There may be a nervous force existing between you and me, and yet we can not cut it and make it bleed, or anything of that kind.

Dr. Wells. While we are on this technical business, I would like to ask Dr. Chappell, what kind of a definition he is going to give to this ulcer where the destruction has proceeded further than the peridental membrane? He speaks of ulceration of the peridental membrane. I did not know that it extended further than the peridental membrane. Then what kind of ulceration is it when it is between the walls of the bone?

Dr. Chappell. Caries of the nerve structure.

Dr. Wells. Suppose it has got to the bone of the membrane, and the bones are not dead, and the abscess forms there?

Dr. Chappell. The peridental membrane or the periosteum?

Dr. Wells. No, sir; the periosteum?

Dr. Chappell. Most generally it is ulceration of the periosteum.

On motion, the subject was passed, and the Association adjourned until 9 o'clock tomorrow morning.—*Indiana State Dental Association.*

---

ARTICLE III.

